# The SapA Protein Is Involved in Resistance to Antimicrobial Peptide PR-39 and Virulence of *Actinobacillus pleuropneumoniae*

**DOI:** 10.3389/fmicb.2017.00811

**Published:** 2017-05-10

**Authors:** Fang Xie, Yalei Wang, Gang Li, Shuanghong Liu, Ning Cui, Siguo Liu, Paul R. Langford, Chunlai Wang

**Affiliations:** ^1^Division of Bacterial Diseases, State Key Laboratory of Veterinary Biotechnology, Harbin Veterinary Research Institute, Chinese Academy of Agricultural SciencesHarbin, China; ^2^Shanghai Veterinary Research Institute, Chinese Academy of Agricultural SciencesShanghai, China; ^3^Section of Paediatrics, Department of Medicine, Imperial College LondonLondon, UK

**Keywords:** *Actinobacillus pleuropneumoniae*, SapA, antimicrobial peptide resistance, PR-39, Virulence

## Abstract

Antimicrobial peptides are essential to the innate immune defense of the mammal against bacterial infection. However, pathogenic bacteria have evolved multiple strategies to resist and evade antimicrobial peptides, which is vital to bacterial survival and colonization in hosts. PR-39 is a linear porcine antimicrobial peptide containing 39 amino acid residues with a high proline content. Resistance to antimicrobial peptide PR-39 has been observed in *Actinobacillus pleuropneumoniae*. However, little is known about the factors required for this resistance. In the present study, PR-39 exposure increased the expression of the *sapA* gene in *A. pleuropneumoniae*. The *sapA* gene, which encodes a putative peptide transport periplasmic protein, was deleted from this bacterium. The Δ*sapA* mutant showed increased sensitivity to PR-39 compared to the wild-type MD12 and complemented PΔ*sapA* strains. However, the Δ*sapA* mutant did not exhibit any alterations in outer membrane integrity. Scanning electron microscopy showed that the Δ*sapA* mutant displayed morphological defects, as indicated by a deformed and sunken shape after PR-39 treatment. In addition, disruption of the SapA protein led to reduced colonization and attenuated virulence of *A. pleuropneumoniae* in the BALB/c mouse model. Collectively, these data suggest that SapA acts as one mechanism for *A. pleuropneumoniae* to counteract PR-39-mediated killing. To the best of our knowledge, this is the first study to show a mechanism underlying antimicrobial peptide resistance in *A. pleuropneumoniae*.

## Introduction

*Actinobacillus pleuropneumoniae* is a Gram-negative bacterial pathogen responsible for porcine pleuropneumonia, which is a highly contagious respiratory disease that causes major economic losses to the swine industry worldwide (Chiers et al., 2010; Bossé et al., 2017). This pathogen mainly causes damage to respiratory tissue, leading to hemorrhagic, fibrinous and necrotic lung lesions (Bossé et al., 2002; Li et al., 2016). The ability of *A. pleuropneumoniae* to adhere to, colonize, and invade its host, and host factors such as innate and adaptive immune responses are crucial to the outcome of this disease (Chiers et al., 2010).

Antimicrobial peptides, also known as host defense peptides, are important components of innate immunity as a first line of defense against bacterial infection (Band and Weiss, 2015). Relative to other mammals, the pig has the most diverse set of cathelicidins (Wessely-Szponder et al., 2010). According to their primary amino acid structures, porcine cathelicidins divide into three subgroups: linear proline-rich cathelicidins (including PR-39, Prophenin 1 and 2), disulfide-rich Protegrins 1–5, and α-helix-rich porcine myeloid antimicrobial peptides (PMAP)-23, PMAP-36, and PMAP-37 (Sang and Blecha, 2009). The proline-rich antimicrobial peptide PR-39 contains 39 amino acid residues with high contents of proline (49%) and arginine (26%) (Zhang et al., 2000). PR-39 manifests antibacterial activity against a variety of Gram-negative bacteria and some Gram-positive bacteria, including multidrug-resistant clinical isolates (Linde et al., 2001). Like other proline-rich peptides, PR-39 kills bacteria without inducing lysis through pore-forming mechanisms. Instead, it translocates across the membrane and disrupts DNA and protein synthesis (Pranting et al., 2008). In addition to its antibacterial activity, PR-39 exerts other important functions, including immunomodulation, wound repair, and the prevention of inflammation during tissue injury (Shi et al., 1996; Veldhuizen et al., 2014).

PR-39 is prominent in tissue of the upper and lower respiratory tract of healthy pigs, and of pigs infected with *A. pleuropneumoniae*, and plays a pivotal role in the innate immune defense of the pig against *A. pleuropneumoniae* infections (Hennig-Pauka et al., 2012). The concentration of PR-39 has been shown to be significantly elevated in bronchoalveolar lavage fluid (BALF) of pigs chronically infected with *A. pleuropneumoniae* (Hennig-Pauka et al., 2006). However, the minimal inhibitory concentration (MIC) of PR-39 for *A. pleuropneumoniae* was 5-fold higher than that of *Escherichia coli*, suggesting the resistance of *A. pleuropneumoniae* to PR-39 (Hennig-Pauka et al., 2006). This resistance observed *in vitro* is consistent with the ability of the pathogen to persist in pig respiratory tissue for long periods. The cause of this resistance is not yet clear. *A. pleuropneumoniae* may have already evolved several strategies to control or evade killing by PR-39 *in vivo*, and the ability to adapt to PR-39 exposure is vital to the pathogenicity of *A. pleuropneumoniae*.

One of the important strategies for bacterial evasion of antimicrobial peptides involves the aid of transporter systems (Band and Weiss, 2015). The Sap transporter system is important for resistance to antimicrobial peptides in several Gram-negative pathogens, including *Haemophilus ducreyi*, non-typeable *Haemophilus influenzae*, and *Salmonella enterica* serovar Typhimurium (Parra-Lopez et al., 1993; Mason et al., 2005; Mount et al., 2010). In general, the Sap transporter consists of five proteins: SapA is a periplasmic solute binding protein, SapB and SapC are permease subunits of the transporter, and SapD and SapF function as ATPase proteins for providing energy to translocate the substrate(s) across the bacterial inner membrane (Parra-Lopez et al., 1993; Mount et al., 2010).

Analysis of the *A. pleuropneumoniae* serovar 5 strain L20 genome sequence (NC_009053.1) reveals the presence of a predicted intact *sap* operon. The nucleotide sequence of *sapA* of *A. pleuropneumoniae* L20 is similar to that of the *sapA* genes of *H. ducreyi* strain 35000HP and non-typeable *H. influenzae* strain 86-028NP, with 68.1 and 53.6% identity, respectively. The amino acid sequence of SapA exhibited 71.1 and 44.4% sequence identity with the SapA protein of *H. ducreyi* strain 35000HP and non-typeable *H. influenzae* strain 86-028NP, respectively. The SapA protein of *A. pleuropneumoniae* has been shown to be expressed *in vivo* during the chronic stage of the disease and is responsible for persistence of *A. pleuropneumoniae* (Baltes et al., 2007). In addition, the *sapF* gene has been reported to be up-regulated in BALF (Lone et al., 2009). Interestingly, PR-39 translocates across the cellular membrane of bacteria via some sort of transport system (Li et al., 2014). It is therefore here hypothesized that the Sap transporter is involved in the resistance of *A. pleuropneumoniae* to PR-39. To test this hypothesis, a nonpolar, unmarked deletion mutation in the *sapA* gene was constructed to investigate the role of SapA protein in PR-39 resistance of *A. pleuropneumoniae*. This is the first demonstration of the mechanism underlying antimicrobial peptide resistance in *A. pleuropneumoniae* and may improve comprehension of the role of the SapA protein in the persistence and pathogenicity of *A. pleuropneumoniae*.

## Materials and methods

### Ethics statement

Animal experiments were approved by Animal Ethics Committee of Harbin Veterinary Research Institute of the Chinese Academy of Agricultural Sciences (CAAS) and carried out in strict accordance with the recommendations of the Animal Ethics Procedures and Guidelines of the People's Republic of China. All efforts were made to minimize animal suffering.

### Bacterial strains and growth conditions

The bacterial strains and plasmids used for this study are described in Table 1. The *A. pleuropneumoniae* strains were cultured in a brain heart infusion (BHI, Difco Laboratories, Detroit, MI, USA) medium supplemented with 10 μg/ml nicotinamide adenine dinucleotide (NAD) (Sigma-Aldrich, U.S.). For culture of *A. pleuropneumoniae* transconjugants (single crossovers), BHI medium was supplemented with 10 μg/ml of NAD and 7 μg/ml of chloramphenicol. *E. coli* ATCC 25922 strain and *S. enterica* ATCC 51741 strain (American Type Culture Collection, ATCC) were cultured in a Luria-Bertani (LB, Difco Laboratories, Detroit, MI, USA) medium. *E. coli* β2155 was grown in LB medium supplemented with 1 mM diaminopimelic acid (DAP) (Sigma-Aldrich, U.S.). All strains were routinely grown at 37°C.

**Table 1:** **Characteristics of bacterial strains and plasmids used in this study**.

**Strains, plasmids, and primers**	**Characteristics or sequence**	**Source or references**
**STRAINS**		
*E. coli* β2155	*thrB1004 pro thi strA hsdS lac*ZΔM15 (F′*lacZ*ΔM15 *lacl*^q^ *traD36 proA*^+^ *proB*^+^)*Δdap*:: *erm* (Erm^r^))*recA:*: *RPA-2-tet*(Tc^r^)::Mu-km (Km^r^) λ*pir*	Dehio and Meyer, 1997
*E. coli* ATCC 25922	*E. coli* serovar O6 isolate	ATCC
*S. enterica* ATCC 51741	*S. enterica* serovar Infantis isolate	ATCC
*A. pleuropneumoniae* ATCC 27090	*A. pleuropneumoniae* serovar 3 isolate	ATCC
*A. pleuropneumoniae* S-8	*A. pleuropneumoniae* serovar 7 clinical isolate from the lung of a dead pig with pleuropneumonia in Heilongjiang province	Lab stock
*A. pleuropneumoniae* MD12	*A. pleuropneumoniae* serovar 5 clinical isolate from the lung of a dead pig with pleuropneumonia in Heilongjiang province	Lab stock
*A. pleuropneumoniae* Δ*sapA*	Unmarked *sapA* gene knockout mutant of *A. pleuropneumoniae* MD12	This work
*A. pleuropneumoniae* PΔ*sapA*	The complemented strain of *A. pleuropneumoniae* Δ*sapA* containing the *sapA* ORF and 675 bp of the upstream region	This work
*A. pleuropneumoniae* Δ*vacJ*	Unmarked *vacJ* gene knockout mutant of *A. pleuropneumoniae* MD12	Xie et al., 2016
**PLASMIDS**		
pEMOC2	Conjugative vector based on pBluescript SK with mob RP4, polycloning site, *Cm^r^*, and transcriptional fusion of the *omlA* promoter with the *sacB* gene	Accession no. AJ868288 (Baltes et al., 2003)
pEMΔ*sapA*	Conjugative vector pEMOC2 with a 570 bp deletion in the *sapA* gene which have a 1.3-kb upstream fragment and 1.3-kb downstream fragment	This work
pGZRS-19	*A. pleuropneumoniae-E. coli* shuttle vector; Ap^r^	West et al., 1995
pGZRS-sapA	pGZRS-19 with a PCR-derived insert containing the *sapA* gene	This work

### Minimum inhibitory concentration (MIC) and minimal bactericidal concentration (MBC) analysis

A microdilution broth method was performed to determine the minimal inhibitory concentration (MIC) of antimicrobial peptide PR-39 according to the broth micro dilution guideline of the Clinical and Laboratory Standards Institute (CLSI, 2013). PR-39 was purchased from AnaSpec (San Jose, CA). Standardized bacterial suspensions of log-phase cultures of *E. coli* ATCC 25922, *S. enterica* ATCC 51741, *A. pleuropneumoniae* strains ATCC 27090, S-8 and MD12 were prepared and diluted to a concentration of 1 × 10^6^ CFU/ml. MIC determinations were performed using commercially sterile 96-well microtiter plates (Costar 3599, U.S.A.). The MIC value was determined as the lowest concentration of PR-39 that prevented visible growth. Then 20 μl of each bacteria-peptide suspension in the 96-well microtiter plates was plated onto LB or BHI agar plates and incubated for 20 h at 37°C. The MBC value was determined as the lowest concentration of PR-39 that showing no visible growth on the plates (Hu et al., 2016).

### *In vitro* growth assays

The *A. pleuropneumoniae* wild-type strain MD12 was grown in 5 ml of BHI medium for 15 h, and then diluted to an optical density at 600 nm (OD_600_) of 0.1. Fresh cultures in 5 ml of BHI medium were supplemented with PR-39 (concentration range 0–0.2 μM) and incubated while shaking at 37°C. Growth was monitored by measuring the OD_600_ values at an interval of 1 h using the Eppendorf BioPhotometer (Eppendorf, Germany).

### RNA isolation and qRT-PCR

For RNA isolation, *A. pleuropneumoniae* MD12 strain was grown to mid-logarithmic phase in 3 ml of BHI medium supplemented with PR-39 (concentration range 0–0.2 μM). The cultures were harvested by centrifugation at 10,000 *g* at 4°C. Total RNA was extracted using RNeasy kit (Qiagen) and cDNA was synthesized using the PrimeScript RT reagent kit (TaKaRa, Japan) according to the manufacturer's instructions. The primers used for analysis of *sapA* expression are listed in Table S1. The cDNA samples were amplified using SYBR Green I (TakaRa). Quantitative real-time polymerase chain reactions (qRT-PCR) were performed in a MicroAmp Optical 96-well reaction plate using a Stratagene Mx3000P system (Agilent Technologies, Germany). Amplification efficiency was evaluated using a standard curve generated by qRT-PCR using the cDNA dilution series with three replicates. The stability of the six housekeeping genes *recF, glyA, rho, tpiA, pykA* (Nielsen and Boye, 2005) and *syp* (Lone et al., 2009) was examined using the program geNorm (Vandesompele et al., 2002). The geometric mean of the best-scoring reference genes *glyA, tpiA*, and *syp* was used to normalize the target gene expression levels. The qRT-PCR experiments were performed in triplicate with three independent biological replicates. Relative expression levels were analyzed by a threshold cycle (ΔΔCt) method to calculate the fold change in gene expression (Pfaffl, 2001).

### Construction of gene deletion mutant

The primers used for the construction of the deletion mutant Δ*sapA* are listed in Table S1. Primers AUF/AUR, and ADF/ADR were used to amplify the two segments flanking the *sapA* gene. Using single-overlap extension PCR (SOE PCR), the fragment with a 570 bp internal in-frame deletion in the *sapA* gene (from nt 24 to 593) was generated, and cloned into the conjugative vector pEMOC2 (Baltes et al., 2003) to produce the plasmid pEMΔ*sapA*. Using *E. coli* β2155 and a single-step transconjugation system (Dehio and Meyer, 1997; Oswald et al., 1999), plasmid pEMΔ*sapA* was used to introduce the *sapA* mutation into the wild-type strain MD12. After two homologous recombination steps, the *A. pleuropneumoniae* Δ*sapA* mutant was verified by sequencing and PCR analyses using AJDF/AJDR primers.

### Complementation of the *A. pleuropneumoniae ΔsapA* mutant

The 2,471 bp PCR product including the entire *sapA* open reading frame (ORF) and 675 bp of the upstream region containing the native promoter was amplified with the primers AHBF/AHBR (Table S1). The PCR reaction was performed under the following conditions: 95°C for 3 min, 30 cycles with 94°C for 30 s, 52 °C for 30 s and 72°C for 2 min, the final extension at 72°C for 8 min. The PCR product was digested with SalI/SacI and ligated to SalI/SacI-digested pGZRS-19 plasmid (West et al., 1995), yielding plasmid pGZRS-sapA. The recombined plasmid pGZRS-sapA was confirmed by DNA sequencing (Comate Bioscience Co., Ltd.) and electroporated into the Δ*sapA* mutant for *trans* complementation. The electroporation conditions were set to 2,500 V, 200 Ω, and 25μF. Transformants were selected on BHI agar containing 20 μg/ml of ampicillin. The complemented mutant strain, verified by colony PCR and DNA sequencing, was designated PΔ*sapA*.

### Bactericidal assays

Bactericidal assays were performed as described previously (Mason et al., 2005). The *A. pleuropneumoniae* strains MD12, Δ*sapA*, and PΔ*sapA* were grown in BHI medium to OD_600_ 0.8. Cells of each strain from the broth cultures were harvested and diluted in PBS (pH 7.4) to a concentration of 10^6^ CFU/ml. The wells of a sterile, polystyrene 96-well microtiter plate (Costar 3599, U.S.A.) were filled with 90 μl of PBS. PR-39 was serially diluted in the wells and each well retained 90 μl of the appropriate concentration (0.5–4 μM) of PR-39. Ten microliters of the bacterial suspension were added to each well, and the plate was incubated for 0.5–3 h at 37°C. Bacteria incubated with PBS served as controls. Serial dilutions of the bacteria were plated on BHI agar. The bactericidal effect was expressed as the percentage of surviving cells, using the bacterial counts obtained with bacteria incubated in PBS as 100%.

### SDS-EDTA sensitivity assay

SDS-EDTA sensitivity assay was performed as described previously (Carpenter et al., 2014). *A. pleuropneumoniae* strains MD12, Δ*sapA*, and PΔ*sapA* were incubated in BHI medium at 37°C with shaking at 180 rpm to OD_600_ 1.0. Each strain was serially diluted with PBS, and 2 μl of these dilutions were spotted in triplicate onto fresh BHI agar plates containing 0.1% SDS and 0.5 mM EDTA. All the plates were incubated overnight at 37°C.

### NPN uptake assay

The 1-N-phenylnaphthylamine (NPN) uptake assay was performed as described previously (Martinez De Tejada and Moriyon, 1993). *A. pleuropneumoniae* strains MD12, Δ*sapA*, and PΔ*sapA* were grown to OD_600_ 0.6 and harvested by centrifugation at 2,500 g for 15 min. Pellets were washed three times and resuspended in 5 mM HEPES buffer (pH 7.2) containing 10 μM NPN (Sigma–Aldrich, USA). NPN uptake into the *A. pleuropneumoniae* Δ*vacJ* mutant was used as a positive control, as *vacJ* encodes VacJ lipoprotein and the membrane permeability of this mutant was increased as described previously (Xie et al., 2016). Fluorescence was measured using the EnVision Multilabel Reader (PerkinElmer, UK), with emission at 420 nm and excitation at 350 nm.

### Scanning electron microscopy

The *A. pleuropneumoniae* strains MD12, Δ*sapA* and PΔ*sapA* were cultivated in BHI medium at 37°C to mid-logarithmic growth phase. Cells of each strain from the broth cultures (10^6^ CFU/ml) were incubated with 4 μM PR-39 for 1 h, and harvested by centrifugation. The cells were washed three times with PBS, and fixed overnight using 2.5% glutaraldehyde at 4°C. Dehydration was performed in upgraded ethanol (washed once with each of 50, 70, 85, 95%, and three times with 100%). Then the samples were dried using a critical point drying method and sputter-coated with gold. The cell morphology of all samples was visualized using a scanning electron microscope (JSM-7500F, JEOL, Japan).

### Mouse *In vivo* experiments

The BALB/c mouse model has been acknowledged as an appropriate one to assess *A. pleuropneumoniae* infection (Chiang et al., 2009; Seo et al., 2013). Specific-pathogen-free, 6-week-old female BALB/c mice (Beijing Vital River Laboratory Animal Co., Ltd.) were purchased from the VitalRiver Laboratories (VRL, Beijing, China). *A. pleuropneumoniae* strains MD12 and Δ*sapA* were cultured in BHI medium at 37°C, and harvested during the mid-exponential phase and washed three times with sterile PBS. A total of 50 mice were randomly divided into 5 groups (*n* = 10/group). Group 1 and group 2 were respectively inoculated intraperitoneally with 100 μl of PBS containing 10^8^ and 10^7^ CFU of MD12. Group 3 and group 4 were respectively inoculated intraperitoneally with 100 μl of PBS containing 10^8^ and 10^7^ CFU of Δ*sapA*. Non-infected mice in the control group were inoculated with 100 μl of sterile PBS (pH 7.4). The health status and the weight of the mice were monitored twice daily for a 14-day period and humane endpoints used to determine if the mice met criteria to be euthanized (Nemzek et al., 2004). These criteria included weight loss >10–15%, lethargy, inability to stand, anorexia or flocked together for more than 6 h. Mice meeting criteria were euthanized by cervical dislocation under isoflurane anesthesia.

### Enumeration of bacterial load in organs

A total of 10 specific-pathogen-free, 6 week-old female BALB/c mice were randomly divided into 2 groups (*n* = 5), and each group was intraperitoneally administered with 5.0 × 10^6^ CFU of the MD12 strain or the Δ*sapA* mutant. Three days post-infection, mice from each group were humanely euthanized and the organs of lung, liver, and kidney were removed aseptically. Samples were weighed, and homogenized using a tissue homogenizer (100 mg weight/ml of PBS). Viable counts in serial dilutions of homogenates were determined following culture on BHI agar plates for 24 h at 37°C.

### Statistical analysis

Statistical analyses were performed using GraphPad Prism version 5.01 (GraphPad Software Inc., U.S.A.). The data are expressed as the means ± standard deviation. The statistical analysis of the data was performed using one-way ANOVA, two-way ANOVA, or the Student's *t*-test. *P*-values less than 0.05 were considered statistically significant.

## Results

### Antibacterial activity of PR-39

To explore the antibacterial activity of PR-39, the MICs of PR-39 for *A. pleuropneumoniae* strains ATCC 27090, S-8, MD12, *E. coli* ATCC 25922 and *S. enterica* ATCC 51741 were measured (Table S2). For *E. coli* and *S. enterica*, the MICs of PR-39 ranged from 0.5 to 1 μM, the MBCs of PR-39 were 1 μM. However, PR-39 had higher MICs for *A. pleuropneumoniae* strains than *E. coli* and *S. enterica*, ranging from 4 to 8 μM. Similarly, the MBCs of PR-39 for *A. pleuropneumoniae* strains were 8 μM, much higher than those of *E. coli* and *S. enterica*. These results showed that *A. pleuropneumoniae* exhibited a certain resistance to PR-39 compared to *E. coli* and *S. enterica*.

### *A. pleuropneumoniae* exposure to PR-39 upregulated expression of the *sapA* gene

**Figure 1:**
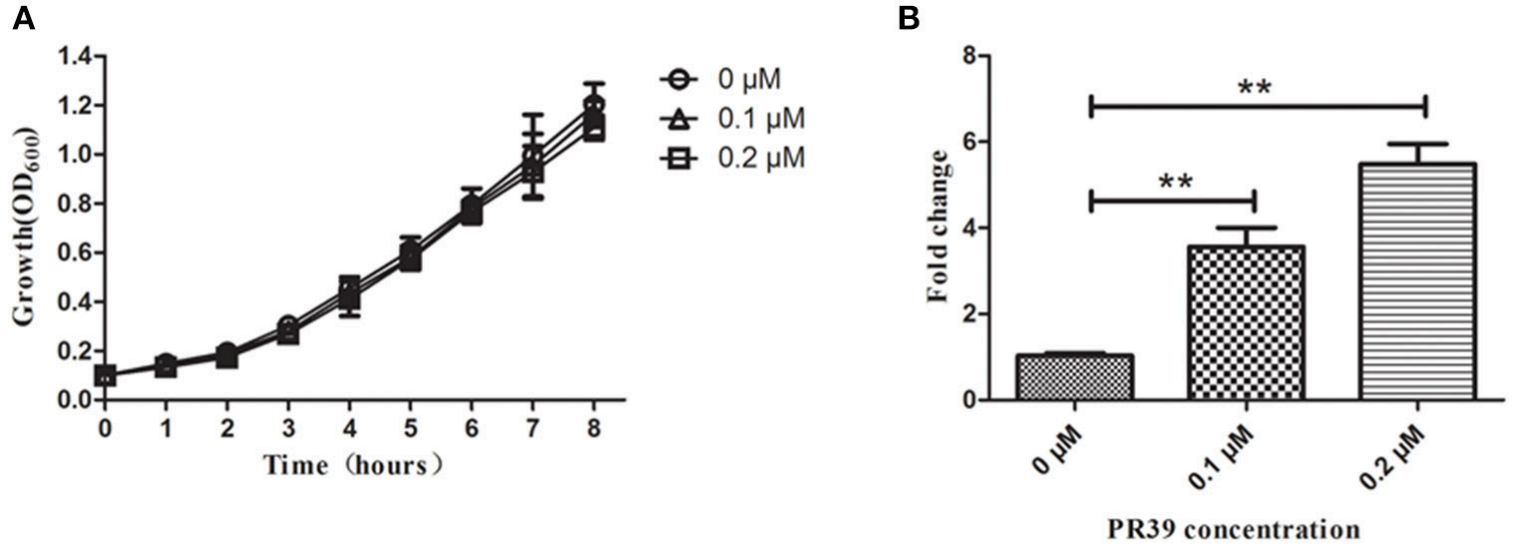
**The expression of ***sapA*** gene of ***A. pleuropneumoniae*** upon exposure to PR-39 by qRT-PCR. (A)** *A. pleuropneumoniae* strain MD12 was grown in the presence of increasing, yet sublethal concentrations of PR-39. **(B)** Transcriptional alteration of *sapA* was examined by quantitative analysis of mRNA expression levels in MD12 when exposed to increasing, yet sublethal concentrations of PR-39. Values represent three independent assays in triplicate ± SD, ^**^p < 0.01.

The expression of the *sapA* gene was analyzed using qRT-PCR in *A. pleuropneumoniae* MD12 when exposed to PR-39. Three housekeeping genes *glyA, tpiA*, and *syp* were selected to normalize *sapA* gene expression levels, and PCR efficiency for each gene was not less than 1.92. When the MD12 strain was exposed to sublethal concentrations of PR39, growth curves were similar to that of untreated bacteria (Figure 1A), but the transcription levels of *sapA* were higher than that of untreated bacteria (Figure 1B). In the presence of increasing concentrations of PR-39, the expression of *sapA* was upregulated in a dose- dependent manner, suggesting *sapA* may contribute to a resistance mechanism in *A. pleuropneumoniae* MD12 against PR-39.

### Construction of *A. pleuropneumoniae ΔsapA* mutant and its complemented strain

**Figure 2:**
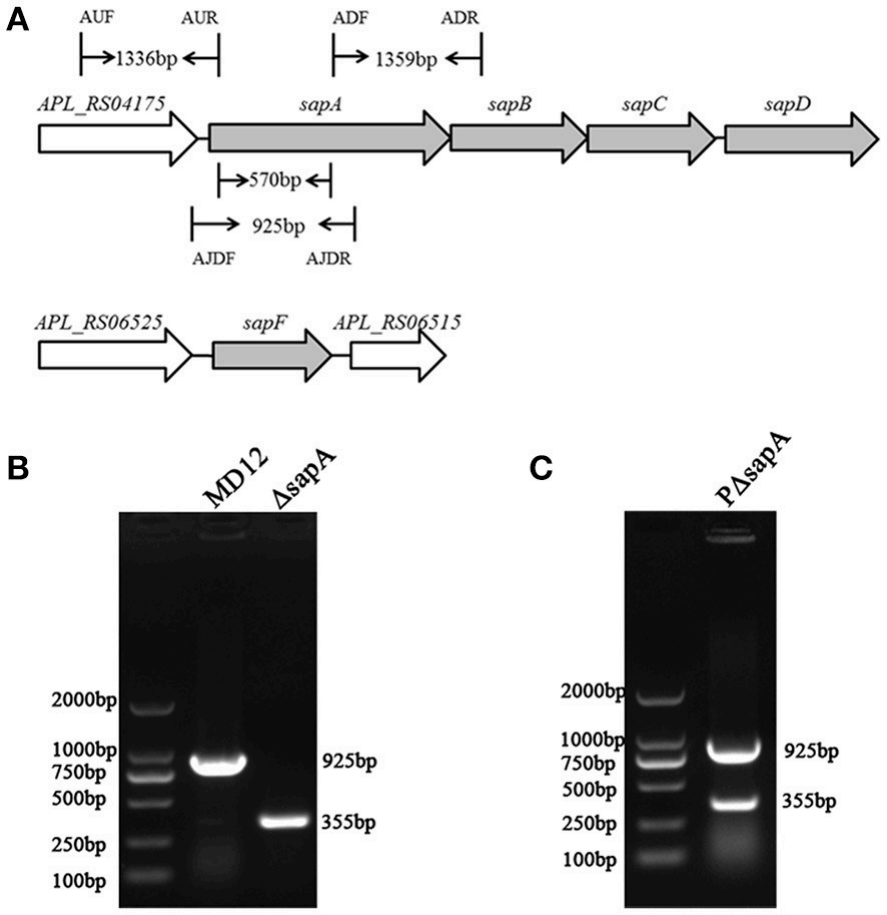
**Chromosomal inactivation of ***A. pleuropneumoniae sapA*** gene. (A)** Schematic representation of the *A. pleuropneumoniae sap* operon locus. The *sapA* gene was inactivated using double-crossover homologous recombination. Binding locations for the primers AUF/AUR and ADF/ADR used to amplify the two flanking regions (1,336 and 1,359 bp, respectively) of the *sapA* gene are shown in the schematic, and primers AJDF/AJDR were here used to identify the *sapA*-deleted mutant (355 bp) and wild-type MD12 strain (925 bp). **(B)** PCR identification of the Δ*sapA* mutant using the primers AJDF/AJDR. **(C)** PCR identification of the complemented strain PΔ*sapA* using the primers AJDF/AJDR.

Analysis of the *A. pleuropneumoniae* L20 genome sequence revealed the presence of an intact *sap* operon (Figure 2A). This operon consists of four genes: *sapA* (APL_RS04170), *sapB* (APL_RS04165), *sapC* (APL_RS04160), *sapD* (APL_RS04155). However, sa*pF* (APL_RS06520) is not linked to the *sapABCD* locus.

To investigate the function of the SapA protein, an in-frame-deletion mutant of *sapA* in *A. pleuropneumoniae* was constructed using double-crossover homologous recombination and confirmed by PCR and DNA sequencing (Figures 2A,B, Supplementary Materials). PCR with primers AJDF/AJDR was used to amplify the 925 bp amplicon from the wild-type MD12 strain, and the 355 bp amplicon from the *sapA* deletion mutant Δ*sapA* (Figure 2B). The Δ*sapA* mutant contains a 570 bp in-frame deletion in the *sapA* gene. The results of qRT-PCR showed that the transcription levels of the downstream genes *sapB, sapC*, and *sapD* were unaffected, confirming that the mutation in Δ*sapA* was nonpolar (Figure S1). The complemented mutant strain PΔ*sapA* was generated using the plasmid pGZRS-sapA, with transformants selected on plates containing ampicillin, and confirmed by PCR (Figure 2C).

### Mutation in *sapA* enhanced sensitivity of *A. pleuropneumoniae* to PR-39

**Figure 3:**
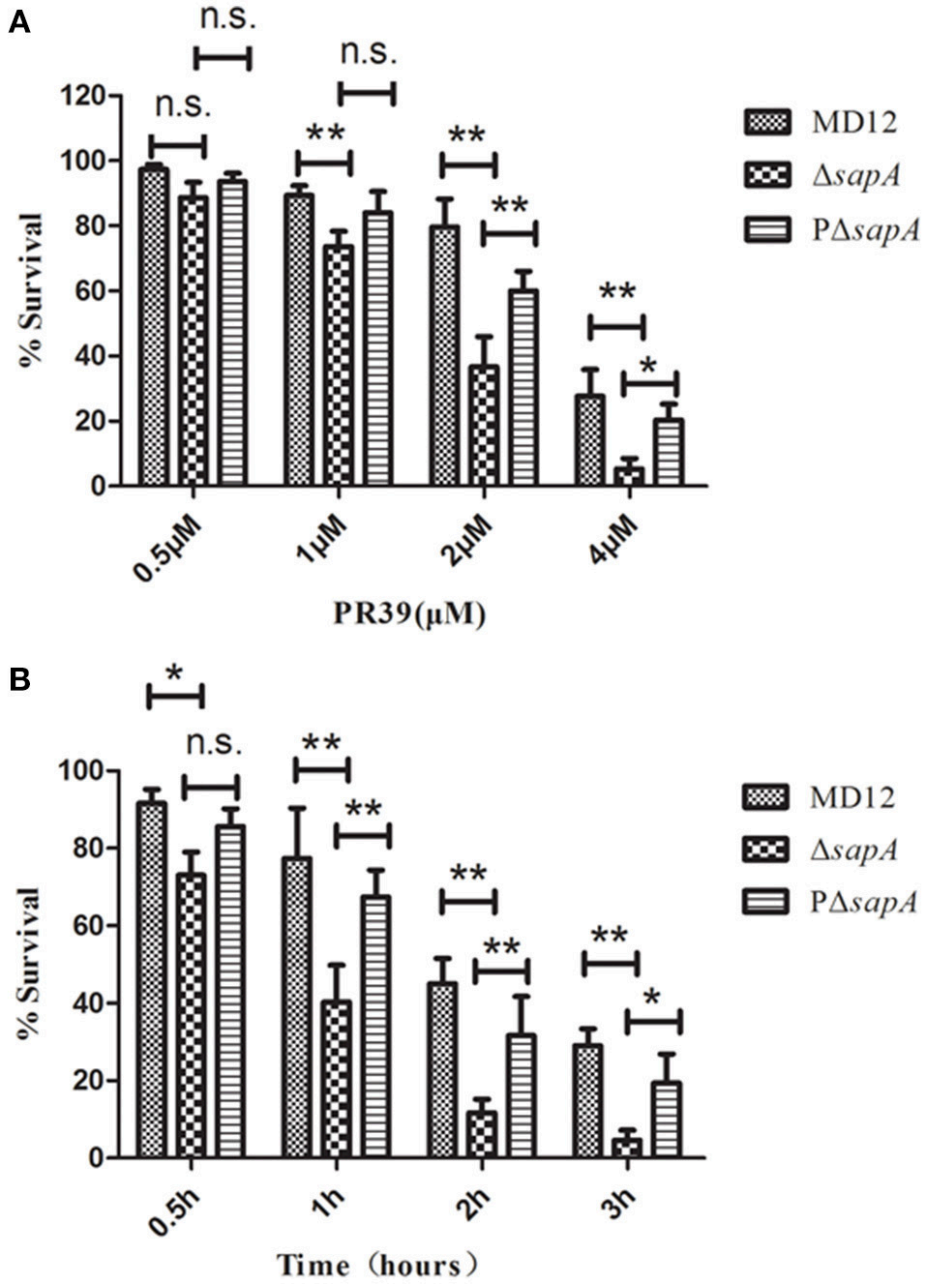
**Sensitivity of the ***sapA*** mutant to PR-39**. MD12, Δ*sapA*, and PΔ*sapA* strains were incubated with **(A)** increasing concentrations of PR-39 for 3 h; and **(B)** 4 μM of PR-39 for increasing periods. Each sample was plated on BHI agar for identification of viable bacteria. Values represent three independent assays in triplicate ± SD, n.s. = not significant, ^*^p < 0.05, ^**^p < 0.01.

To determine whether the SapA protein has a role in the survival of *A. pleuropneumoniae* upon exposure to the antimicrobial peptide PR-39, the MD12, Δ*sapA*, and PΔ*sapA* strains were tested in a bactericidal assay. Cells of each strain were incubated with specific concentrations of PR-39 for 3 h. The results showed that the mutant devoid of *sapA* was significantly more sensitive to PR-39 over a concentration range of 0.5–4 μM than was the isogenic wild type strain (Figure 3A). In addition, following the elongation of treatment time, the sensitivity of Δ*sapA* to PR-39 was increased (Figure 3B). Trans-complementation with *sapA* expressed on pGZRS-19 partially restored resistance to PR-39. These findings indicate that the *A. pleuropneumoniae* SapA protein is required for the bacterium's resistance to the PR-39.

To exclude the possibility of impaired outer membrane integrity due to the deletion of the *sapA* gene, the sensitivity of the MD12, Δ*sapA*, and PΔ*sapA* strains to SDS-EDTA was analyzed. As shown in Figure S2A, all these strains did not exhibit sensitivity to SDS-EDTA. In addition, the outer membrane integrity of each strain was further evaluated using the fluorescent probe NPN, which exhibits fluorescence weakly in aqueous but strongly in hydrophobic environments (Lee et al., 2015). In Figure S2B, no significant difference in uptake of NPN was observed between MD12, Δ*sapA*, and PΔ*sapA*, while NPN fluorescence was significantly higher in the Δ*vacJ* mutant whose membrane permeability was increased as described previously (Xie et al., 2016). These data indicated that the *sapA* gene deletion did not cause alterations in the outer membrane integrity in *A. pleuropneumoniae*.

### Morphology of *A. pleuropneumoniae ΔsapA* upon exposure to PR-39

**Figure 4:**
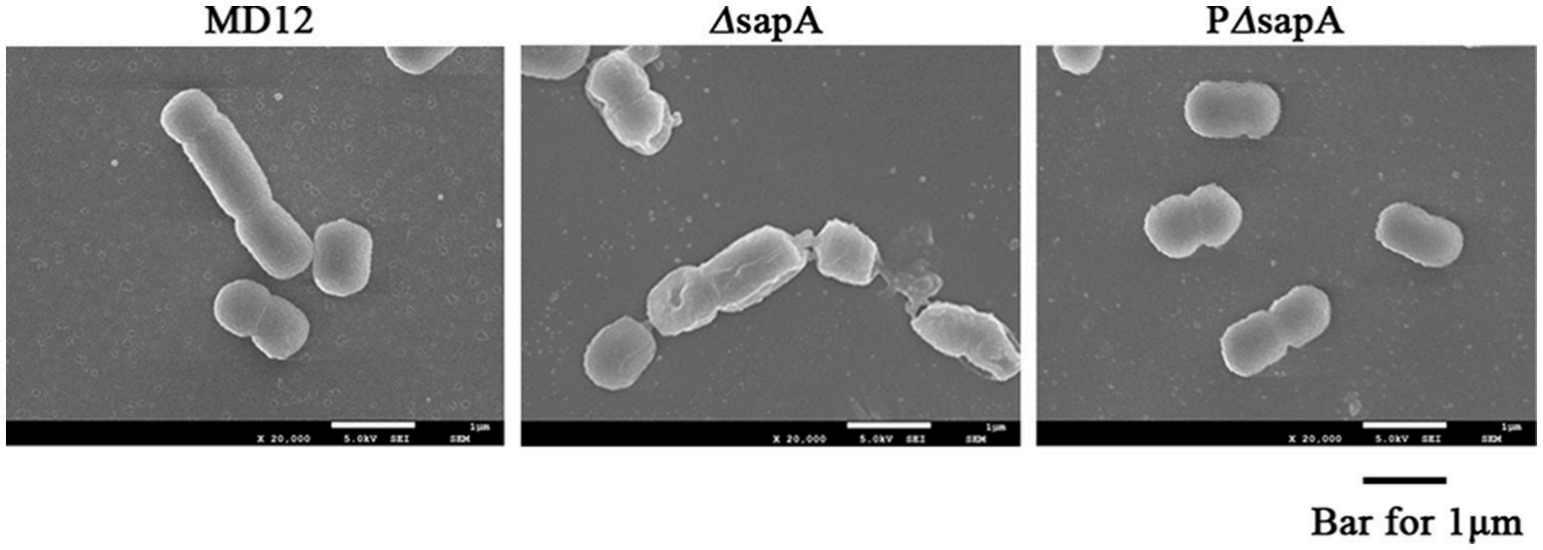
**Scanning electron microscopy of ***A. pleuropneumoniae*** strains treated with PR-39**. MD12, Δ*sapA*, and PΔ*sapA* in the mid- logarithmic phase were harvested and treated with 4 μM PR-39 for 30 min. Scale bar, 100 nm. The cells of Δ*sapA* displayed a relatively deformed cell shape compared to that of the wild-type MD12 strain and the complemented strain PΔ*sapA*.

To further confirm the increased sensitivity of the Δ*sapA* strain to PR-39, the morphology of the MD12, Δ*sapA*, and PΔ*sapA* strains treated with PR-39 was assessed using scanning electron microscopy. After PR-39 treatment, a significant morphological variation was observed among the MD12, Δ*sapA*, and PΔ*sapA* strains (Figure 4). MD12 displayed a smooth surface, which is typical of this *A. pleuropneumoniae* strain (Figure 4). However, when exposed to PR39, cells of the Δ*sapA* mutant had an irregular and crinkled appearance and a sunken shape compared to that of the wild-type MD12 and complemented PΔ*sapA* strains (Figure 4). These data indicated that the *A. pleuropneumoniae* SapA protein functions, at least to some extent, to protect this pathogen from the lethal effects of PR-39.

### Loss of *sap*A attenuates the virulence of *A. pleuropneumoniae* in the BALB/c mouse model

**Figure 5:**
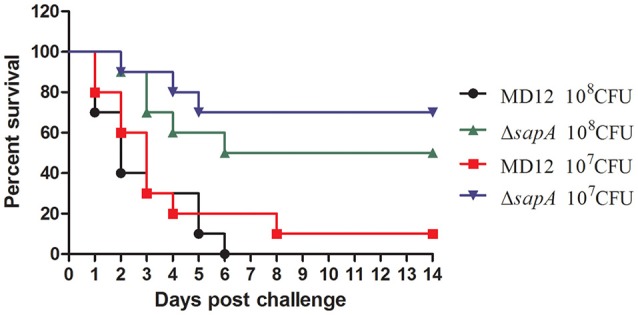
**Survival of mice following intraperitoneally challenge with ***A. pleuropneumoniae*** strains**. Percentage of surviving mice after challenged with 1 × 10^8^ or 1 × 10^7^ CFU of MD12 or Δ*sapA* strain.

To address whether *sapA* deletion affected the virulence of *A. pleuropneumoniae*, BALB/c mice were inoculated intraperitoneally with wild type strain MD12 and the Δ*sapA* mutant at various doses. The MD12 strain gave rise to a higher mortality rate than Δ*sapA* (Figure 5), which suggested that the deletion of *sapA* attenuates the virulence of *A. pleuropneumoniae*.

**Figure 6:**
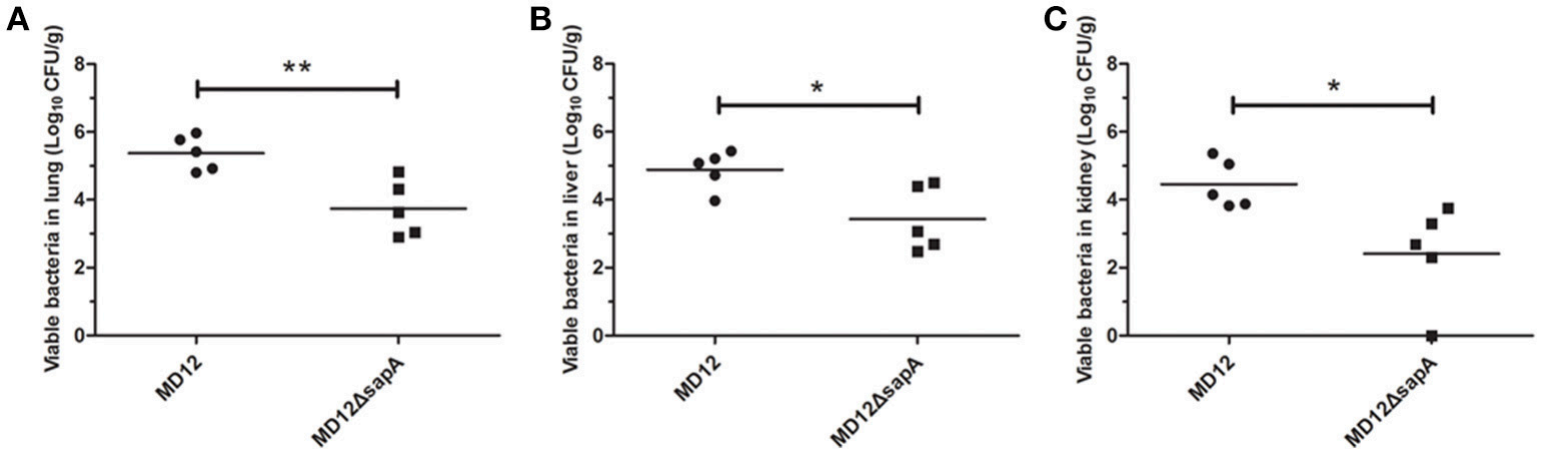
**Bacterial loads in organs from BALB/c mice infected with ***A. pleuropneumoniae*****. Mice were infected with the WT MD12 or Δ*sapA* strain, and bacterial loads in **(A)** lung, **(B)** liver, **(C)** kidney were examined 3 days post infection. The data shown are the means of bacterial colonies from five mice, and error bars indicate standard deviations. Data presented are the mean values and standard deviations of 5 biological replicates, ^*^p < 0.05, ^**^p < 0.01.

The capacity of the MD12 and Δ*sapA* strains to colonize mice was then tested. The *A. pleuropneumoniae* load in tissues of systemically infected mice was determined by culturing the lungs, livers, and kidneys homogenates 3 days post-infection. As shown in Figure 6, the viable counts in lung were significantly decreased in the Δ*sapA* mutant-infected mice compared with the WT-infected mice (*P* < 0.01). Similarly, significant differences (*P* < 0.05) in bacterial loads were also found between the MD12-inoculated and Δ*sapA*-inoculated mice in livers and kidneys (Figure 6). Taken together, the results showed that the Δ*sapA* mutant of *A. pleuropneumoniae* displayed a reduced ability to colonize BALB/c mice.

## Discussion

Antimicrobial peptides are an essential part of innate immune defenses that inhibit pathogen infection and contribute to clearance of bacterial colonization (Band and Weiss, 2015). Upon encountering invasive pathogens, hosts can generate the specific innate immune signaling events to induce production of specific antimicrobial peptides in response to the invasion of pathogens (Plichta et al., 2012). However, to adapt to the environments of elevated antimicrobial peptides, bacteria have evolved multiple countermeasures to resist and evade antimicrobial peptide-mediated killing (Band and Weiss, 2015). Resistance to porcine antimicrobial peptides is vital to survival and colonization of *A. pleuropneumoniae* in host environments (Hennig-Pauka et al., 2006). However, little is known about the factors required for this resistance. This study demonstrated that the putative peptide transport periplasmic protein SapA of *A. pleuropneumoniae* is involved in resistance to PR-39-mediated killing. This is the first study to show an antimicrobial peptide resistance mechanism in *A. pleuropneumoniae*.

PR-39 has been shown to be essential to the innate immune defense of the pig against *A. pleuropneumoniae* infection (Hennig-Pauka et al., 2012). In this study, the MICs of *E. coli* and *A. pleuropneumoniae* isolates were measured in the laboratory, and an MIC of 1 μM was determined for *E. coli*, but 4–8 μM for *A. pleuropneumoniae* strains. This finding is in accordance with the previous report by Hennig-Pauka et al. suggesting innate resistance of *A. pleuropneumoniae* to PR-39 (Hennig-Pauka et al., 2006). This resistance contributes to the promotion of *A. pleuropneumoniae* survival and colonization in the host for extended periods of time. Most notably, the mode of action of PR-39 killing bacteria does not involve the formation of pores, but translocation across the membrane via some sort of transport system and the targeting of intracellular molecules (Li et al., 2014). Thus, we hypothesized that certain peptide transport systems would be required for resistance of *A. pleuropneumoniae* to PR-39. Analysis of the *A. pleuropneumoniae* L20 genome sequence revealed the presence of an intact *sap* operon (Figure 2A). This operon consists of four genes, but does not contain the *sapF* gene: *sapA* (APL_RS04170), which encodes a putative periplasmic binding protein; *sapB* (APL_RS04165) and *sapC* (APL_RS04160), which encode putative permease components; and *sapD* (APL_RS04155) which encodes the ATPase components. The unlinked sa*pF* (APL_RS06520) is predicted to encode the ATPase component of this transporter. The peptide transport periplasmic protein SapA, which has been found to be expressed *in vivo* during the chronic stage of *A. pleuropneumoniae* infection (Baltes et al., 2007), has drawn considerable attention. Additionally, the *sapF* gene has been reported to be up-regulated in *A. pleuropneumoniae* when grown in pig BALF (Lone et al., 2009). The results of the present study showed that inactivation of the *sapA* gene significantly enhanced sensitivity of *A. pleuropneumoniae* to PR-39, indicating that the SapA protein is required for *A. pleuropneumoniae* resistance to the PR-39 (Figures 3, 4).

Until now, several transporter systems have been shown to mediate resistance against antimicrobial peptides. Mutation in the *yejF* gene of *S. enterica* from the *yejABEF* operon encoding an ATP-binding cassette (ABC) peptide import system, reduced resistance to polymyxin B, human defensin (HBD)-1 and HBD-2 (Eswarappa et al., 2008). Additionally, in the pathogens *Neisseria meningitidis* and *H. ducreyi*, a periplasmic membrane fusion protein MtrC actively transports cathelicidin LL-37 out of the bacterial membrane to promote resistance to LL-37 (Tzeng et al., 2005; Rinker et al., 2011). Furthermore, the Sap transporter was also demonstrated to contribute to antimicrobial peptide resistance in other Gram-negative species. In non-typeable *H. influenzae*, mutation in the *sapA* gene led to reduced resistance to the chinchilla beta defensin 1 (cBD-1) (Mason et al., 2005). The Sap transporter in *H. ducreyi* confers resistance to LL-37, but not to α-or β-defensins (Mount et al., 2010). In *Vibrio fischeri*, SapA did not confer resistance to any of the eight tested antimicrobial peptides but was implicated in *in vitro* growth and *in vivo* colonization (Lupp et al., 2002). The present study showed that the SapA protein rendered *A. pleuropneumoniae* resistant to the porcine antimicrobial peptide PR-39 (Figures 3, 4). Taken together, these studies suggested that the SapA protein served multiple functions to satisfy the requirements of different bacterial species over the course of infection.

*In vivo* colonization by *A. pleuropneumoniae* is a complicated process, during which the evasion from the host innate immune plays an important role. Another goal of this study was to clarify whether SapA is essential for *A. pleuropneumoniae* colonization and pathogenicity in a mouse model. The data presented in this study showed that the Δ*sapA* mutant displayed an attenuated virulence and reduced bacterial colonization, compared with wild type strain (Figures 5, 6). These results may be explained by the decreased ability of the Δ*sapA* mutant to resist PR-39 mediated killing *in vivo*. This is highly consistent with the data presented by a previous study which showed that the SapA protein was required for colonization and virulence of non-typeable *H. influenzae* in a chinchilla model (Mason et al., 2005). It is clear from these data that the *sapA* gene product is involved in colonization and virulence in *A. pleuropneumoniae* infection.

The lung is the major target of *A. pleuropneumoniae* infection (Bossé et al., 2002; Chiers et al., 2010). PR-39 expression has been reported to be increased in BALF and epithelial lining fluid (ELF) after *A. pleuropneumoniae* infection of pigs (Hennig-Pauka et al., 2006). The concentration of PR-39 in BALF ranged from 0.4 to 75.9 nM and concentrations in ELF are approximately 6- to 40-fold higher than in BALF in infected pigs, which are lower than the MICs of *A. pleuropneumoniae* strains. At sub-inhibitory concentrations, PR-39 in combination with other antimicrobial factors in the lower respiratory tract may result in a synergistic antimicrobial effect against *A. pleuropneumoniae*. The sub-inhibitory concentrations of PR-39 might also let *A. pleuropneumoniae* adapt to the host environment and is in accordance with the *in vivo* observation that *A. pleuropneumoniae* persists in respiratory epithelium of pigs for extended periods. In addition to its antimicrobial properties, PR-39 is also involved in many other biological processes, such as chemotaxis of neutrophils, promotion of wound healing, and inhibition of apoptosis (Shi et al., 1996; Veldhuizen et al., 2014). In *A. pleuropneumoniae* chronic infection, the involvement of PR-39 in these biological processes might be its primary role.

In conclusion, this study demonstrates that the SapA protein in *A. pleuropneumoniae* promotes resistance to antimicrobial peptide PR-39, and it is the first mechanism of antimicrobial peptide resistance identified in *A. pleuropneumoniae*. In addition, disruption of the SapA protein led to reduced colonization and attenuated virulence of *A. pleuropneumoniae* in the BALB/c mouse model. This has shed light on the role of SapA protein in the pathogenicity of *A. pleuropneumoniae*. Of note, though the *sapA* deletion increased sensitivity of *A. pleuropneumoniae* to PR-39, the MIC of Δ*sapA* was still more than that of *E. coli* (data not shown), indicating the presence of other PR39 resistance mechanisms in *A. pleuropneumoniae*. Future studies should include attempts to determine whether other proteins, such as secreted proteases or two-component systems, participate in the resistance to PR39, and unravel the mechanism of detection and signal transduction that takes place when *A. pleuropneumoniae* encounters PR-39.

## Author contributions

FX and CW designed the experiments, FX and GL conducted experiments, YW, ShL, and NC performed the experiments, FX and YW analyzed the data and drafted the manuscript, CW, PL, and SiL finalized the manuscript. All authors read and approved the final manuscript.

### Conflict of interest statement

The authors declare that the research was conducted in the absence of any commercial or financial relationships that could be construed as a potential conflict of interest.

## Supplementary material

Figure S1 **Transcriptional levels of downstream genes of ***sapA*** in MD12 and Δ***sapA*** strains**. Transcriptional levels of *sapB, sapC, sapD* genes were examined by qRT-PCR. Values represent two independent assays in triplicate ±SD.

Figure S2 **The outer membrane integrity of ***A. pleuropneumoniae***. (A)** SDS-EDTA sensitivity assay. Cultures were grown until mid-log phase, and 2 μl of each dilution, indicated on the left, was spotted in triplicate onto BHI agar plate supplemented with 0.1% SDS and 0.5 mM EDTA. **(B)** NPN uptake assay. Changes in fluorescence following the addition of the hydrophobic fluorescent probe NPN for the MD12, Δ*sapA*, PΔ*sap*, and Δ*vacJ* strains are shown. Values represent two independent assays in triplicate ±SD, n.s. = not significant, ^**^*p* < 0.01.

Table S1 **Primers used in this study**.

Table S2 **Minimum inhibitory concentrations and minimal bactericidal concentration of PR-39 for ***E. coli, S. enterica***, and ***A. pleuropneumoniae*** strains**.
