# Supplementary figures and images for: The SapA Protein Is Involved in Resistance to Antimicrobial Peptide PR-39 and Virulence of Actinobacillus pleuropneumoniae
Source: Front Microbiol. 2017 May 10;8:811. doi: 10.3389/fmicb.2017.00811 (PMC5423912; doi:10.3389/fmicb.2017.00811)

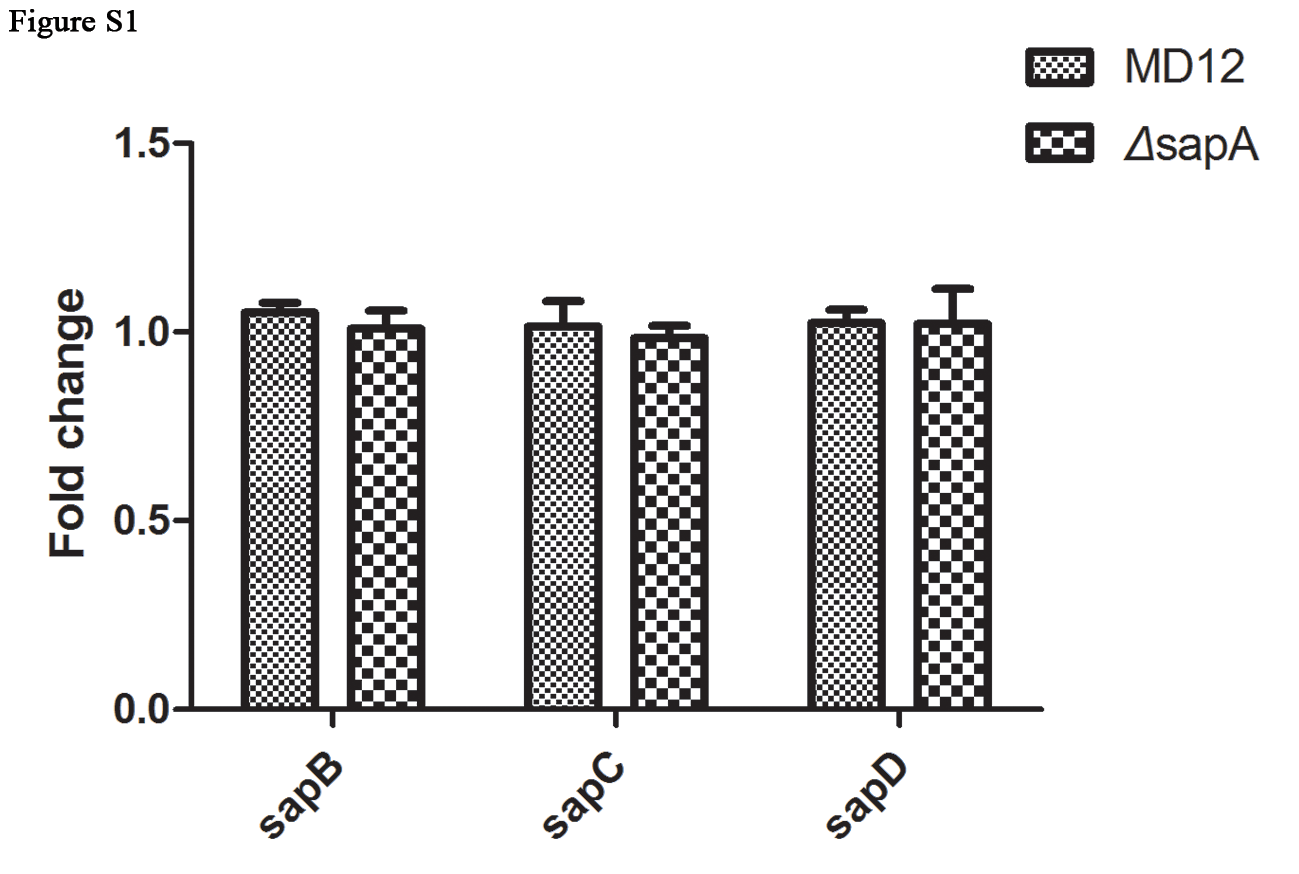

Supplement: Figure S1 — Transcriptional levels of downstream genes of sapA in MD12 and ΔsapA strains. Transcriptional levels of sapB, sapC, sapD genes were examined by qRT-PCR. Values represent two independent assays in triplicate ±SD. [file Image1.TIF]

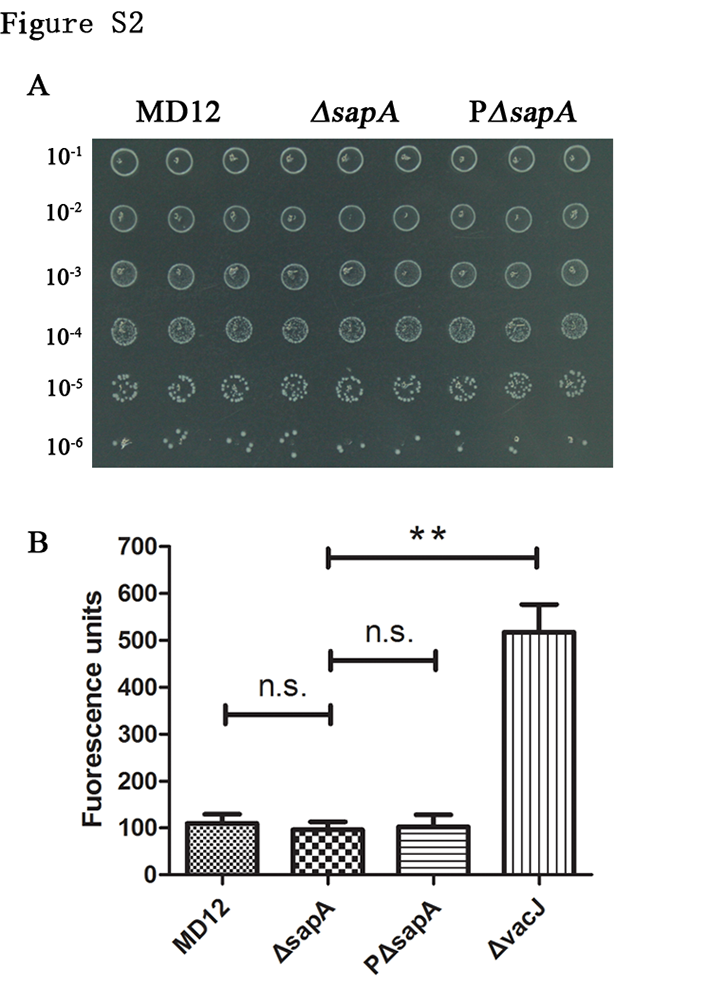

Supplement: Figure S2 — The outer membrane integrity of A. pleuropneumoniae. (A) SDS-EDTA sensitivity assay. Cultures were grown until mid-log phase, and 2 μl of each dilution, indicated on the left, was spotted in triplicate onto BHI agar plate supplemented with 0.1% SDS and 0.5 mM EDTA. (B) NPN uptake assay. Changes in fluorescence following the addition of the hydrophobic fluorescent probe NPN for the MD12, ΔsapA, PΔsap, and ΔvacJ strains are shown. Values represent two independent assays in triplicate ±SD, n.s. = not significant, **p < 0.01. [file Image2.TIF]
