# Supplementary material for: The SapA Protein Is Involved in Resistance to Antimicrobial Peptide PR-39 and Virulence of Actinobacillus pleuropneumoniae
Source: Front Microbiol. 2017 May 10;8:811. doi: 10.3389/fmicb.2017.00811 (PMC5423912; doi:10.3389/fmicb.2017.00811)
Supplement: Table S1 — Primers used in this study. [file Table1.DOCX]

**Table S1**

**Primers used in this study**

| Primer | Sequences (5’–3’) | Source or reference |
| --- | --- | --- |
| AUF | 5' GCGTCGACGTTCGATGCGTTCGAAAGTG 3', upstream primer with internal SalI site (underlined) | This work |
| AUR | 5' GCGAAACC**GCTTGTCG**CATACAAGAAACC 3', downstream primer with reverse complement sequence(underlined) of sequence in bold from primer ADF |  |
| ADF | 5' CGACAAGC**GGTTTCGC**CCGATCAATCC 3', upstream primer with reverse complement sequence(underlined) of sequence in bold from primer AUR | This work |
| ADR | 5' TTGCGGCCGCCAATACCGCCAAACAAAGC 3', downstream primer with internal NotI site (underlined) |  |
| AJDF | 5' ATACGGCATTGGGAAATAAGG -3', upstream primer for identification of △*sapA* mutant | This work |
| AJDR | 5' CGGCAAAGCGACTACATCAC -3', downstream primer for identification of △*sapA* mutant |  |
| AHBF | 5' TTGTCGACTTTGAATATGCTAACGGCG 3', upstream primer with internal *Sal*I site (underlined) | This work |
| AHBR | 5' CGAGCTCATTCAAAGGATCGCGTAGTAAG 3', downstream primer with internal *Sac*I site (underlined) |  |
| recFF | TATGCCGAGATTCTTGCTCA | (Nielsen and Boye, 2005) |
| recFR | AATTTAAGCTGCCCACGAGA |  |
| glyAF | CAAGCGAATGCAGCTGTTTA | (Nielsen and Boye, 2005) |
| glyAR | CTGTGATGCCGTAGAGGACA |  |
| rhoF | AATACCGTGACGCCTGTTTC | (Nielsen and Boye, 2005) |
| rhoR | ACTAATGCCGTCGCGATAAT |  |
| tpiAF | CTACGAACCGATTTGGGCTA | (Nielsen and Boye, 2005) |
| tpiAR | CCGCCGTATTGGATAATCAC |  |
| pykAF | GTACGGATGCGGTAATGCTT | (Nielsen and Boye, 2005) |
| pykAR | ACCTTCCATACGGTGACGAG |  |
| sypF | AAGAAACGCCGAATGATGCACAGG | (Lone et al., 2009) |
| sypR | ACACCTCGATAGCACCACCTTTGT |  |
| sapAF | CGCCTCGTATTCCGCAAG | This work |
| sapAR | CGTAAACCATTCCGTAGCAT |  |
| sapBF | ATGTCTTTCCCGCCACCG | This work |
| sapBR | ATAGGGAGAATCCGCCAAG 3 |  |
| sapCF | GGTACGGCAAGAGTTGAAGAGA | This work |
| sapCR | TCCAAGACCGCAATCACGAA |  |
| sapDF | AGCGACCACGCAGCTACAGAT | This work |
| sapDR | ACCGAGGTATAAGGATGAAACG |  |
