# Supplementary material for: The SapA Protein Is Involved in Resistance to Antimicrobial Peptide PR-39 and Virulence of Actinobacillus pleuropneumoniae
Source: Front Microbiol. 2017 May 10;8:811. doi: 10.3389/fmicb.2017.00811 (PMC5423912; doi:10.3389/fmicb.2017.00811)
Supplement: Table S2 — Minimum inhibitory concentrations and minimal bactericidal concentration of PR-39 for E. coli, S. enterica, and A. pleuropneumoniae strains. [file Table2.DOCX]

**Table S2**

**Minimum inhibitory concentrations and minimal bactericidal concentration of PR-39 for *E. coli*, *S. enterica* and *A. pleuropneumoniae* Strains**

|  | MIC（μM） | MBC（μM） |
| --- | --- | --- |
| *E. coli* ATCC 25922 | 1 | 1 |
| *S. enterica* ATCC 51741 | 0.5 | 1 |
| *A. pleuropneumoniae* ATCC 27090 | 4 | 8 |
| *A. pleuropneumoniae* S-8 | 4 | 8 |
| *A. pleuropneumoniae* MD12 | 8 | 8 |
