# Supplementary material for: The SapA Protein Is Involved in Resistance to Antimicrobial Peptide PR-39 and Virulence of Actinobacillus pleuropneumoniae
Source: Front Microbiol. 2017 May 10;8:811. doi: 10.3389/fmicb.2017.00811 (PMC5423912; doi:10.3389/fmicb.2017.00811)
Supplement: Supplementary file 5 [file Presentation1.PDF]

>DNA sequence amplified with primers AJDF/AJDR from MD12
atacggcattgggaaataaggaaagactatggtttcttgtatgcgacaagcggtcggttttatccgctttttgcaattttagcga
tgattccgaccgcttatagtgcgcctcgtattccgcaagcgttattggataacagcttgatttattgtaccagcgtatccggtttc
agctttaatccgcaaaaagcggacgtcggcaccaatatgaatgtggtgacggaacaaatttacgataagttgttcgagtttga
cgcagcgacaaattccttaaaaccgaaactggtcgaaagttatcagattagcgaagacggaaaagtcattaccttaaaatta
cgtcgtaaagtcgcctttcatgctacggaatggtttacgccgactcgtccgtttaatgcggaagacgtggtttttcattgaaca
gaatgatcgggaatgtcgaagagcttcccgctttggattttaatgaagacggtcagagagaagcattccatcagaatcagtat
tatgcttatcaatttaaagcgaatttggcgcattatccttattttgaaagcatcgccttaaaaaataaaatcgagcgtatttcggca
gtaaacgactatacggtcaagattcatttggtttcgcccgatcaatccgttttagcgcatttggcaagccaatatgcggttatttt
atcgaaagaatacgctttacaattaaatgcggatgaaaacctcgcacaactagatttattaccggttggaaccggtgtttatca
actgagcaattatgtacagaatgaatatgtgcgtttaaaaccgcatcctaaatattggggcaaaaaagccaatattgaaaatat
ggtggtggatgtgtccagtaatgatacggggcgtatggcaaaatatttaaacggtgagtgtgatgtagtcgctttgccg

>DNA sequence amplified with primers AJDF/AJDR from *∆sapA*
atacggcattgggaaataaggaaagactatggtttcttgtatgcgacaagcggtttcgcccgatcaatccgttttagcgcattt
ggcaagccaatatgcggttattttatcgaaagaatacgctttacaattaaatgcggatgaaaacctcgcacaactagatttatta
ccggttggaaccggtgtttatcaactgagcaattatgtacagaatgaatatgtgcgtttaaaaccgcatcctaaatattggggc
aaaaaagccaatattgaaaatatggtggtggatgtgtccagtaatgatacggggcgtatggcaaaatatttaaacggtgagt
gtgatgtagtcgctttgccg
